# Evaluating survival outcomes and treatment recommendations in resectable gastric cancer

**DOI:** 10.1038/s41598-024-82807-8

**Published:** 2025-01-22

**Authors:** Saad Sabbagh, Iktej Singh Jabbal, María Herrán, Mohamed Mohanna, Sindu Iska, Mira Itani, Barbara Dominguez, Kaylee Sarna, Zeina Nahleh, Arun Nagarajan

**Affiliations:** 1 Department of Hematology-Oncology, Maroone Cancer Center, Cleveland Clinic Florida, Weston, FL 33331 USA; 2 Department of Internal Medicine, Advent Health Sebring, Sebring, FL USA; 3 Department of Clinical Research, Cleveland Clinic Florida, Weston, FL USA

**Keywords:** Gastric cancer, Gastroesophageal junction cancer, Survival outcomes, Combination treatment, Treatment trends., Gastric cancer, Cancer therapy

## Abstract

No consensus exists on the optimal therapy for resectable gastric cancer (GC) and gastroesophageal junction (GEJ) tumors, including the effectiveness of chemoradiotherapy versus perioperative chemotherapy (PC). Our study aimed to compare overall survival (OS) outcomes associated with the recommended treatment modalities for GC and GEJ tumors and evaluate treatment trends from 2010 to 2020. A national registry cohort identified patients with ≥ cT2 nonmetastatic GC and GEJ cancer. Treatment modalities were classified as neoadjuvant chemotherapy (NC), neoadjuvant chemoradiotherapy (NCR), PC, adjuvant chemotherapy (AC), and adjuvant chemoradiation (ACR). Kaplan-Meier curve and multivariable Cox regression models evaluated factors associated with OS. A cohort of 7665 patients were included. Patients who received PC had the highest OS (median 86.80 months, 95% CI 73.40-NE), while chemoradiotherapy in the neoadjuvant and adjuvant settings had worse OS than PC and NC (NCR median 47.15 months, 95% CI 44.58–52.27, and ACR median 52.67 months, 95%CI 42.78–63.93). The Cox proportional hazards model showed that NCR and NC had worse survival than PC (HR 1.74, 95% CI 1.50–2.02, *p* < 0.001 and HR 1.26, 95% CI 1.10–1.44, *p* = 0.0008, respectively). Additionally, the most utilized modality during 2020 was NC (35.8%), followed by PC (28.0%) and NCR (24.9%). The utilization of PC and NC had the most substantial rise between 2010 and 2020, increasing by 11.0%. The study demonstrates the association of PC with improved OS outcomes for nonmetastatic GC and GEJ tumors. Therapies combining radiation with chemotherapy and extended lymph node dissection correlated with a worse prognosis compared to PC and NC. Despite the association with improved outcomes, national data reveals low utilization rates for PC.

**Supplementary Information:**

The online version contains supplementary material available at 10.1038/s41598-024-82807-8.

## Introduction

Gastric cancer (GC) is among the most frequently diagnosed and lethal cancers worldwide^1^. In 2020, approximately 1.1 million new cases were diagnosed, and more than 700,000 GC deaths were recorded globally^2^. Prognosis remains poor for regional and distant disease, with a 38% decrease in the 5-year survival between localized and regional stages (72–33%)^3^. Due to the disease’s aggressivity and the high risk of recurrence ranging between 40 and 60%^4^, combination therapy, including chemotherapy, radiation, and surgery, has been incorporated as the standard of care for nonmetastatic stage > IB disease^5,6^.

Throughout the last two decades, different therapeutic modalities have been described in GC trials, including neoadjuvant chemotherapy (NC), neoadjuvant chemoradiotherapy (NCR), perioperative chemotherapy (PC), adjuvant chemoradiation (ACR), and adjuvant chemotherapy (AC)^7^. While PC is recommended with the highest level of evidence based on the results of the notable FLOT-4 and the MAGIC trials^8,9^, relevant questions arise about the tolerability of PC given the high incompletion rates in both trials. Moreover, contemporary literature lacks head-to-head trials comparing the superiority of the treatment modalities described, and the role of chemoradiation in the neoadjuvant and adjuvant settings in comparison to PC is yet to be established. Significant disagreement also exists on managing gastroesophageal junction (GEJ) tumors. These tumors have frequently been grouped with either esophageal or gastric cancers in several pivotal trials and have rarely been evaluated as distinct entities^8,9^.

As a result, the plethora of treatment options among patients with gastric and GEJ cancer have resulted in ambiguity in clinical practice, and national treatment patterns over the last decade have rarely been explored. Therefore, this study compared overall survival (OS) outcomes among different treatment modalities for gastric and GEJ tumors using the National Cancer Database (NCDB) data between 2010 and 2020. We also evaluated the prevalence and changing trends of the treatment modalities utilized over this period.

## Methods

### Data source

This is a retrospective cohort study using the NCDB from 2010 to 2020. The NCDB is a national registry that captures approximately 70% of all newly diagnosed cancer cases in the United States (US) and includes de-identified demographic, clinical, and pathological data from over 1,500 cancer institutions. The principal investigator downloaded the gastric cancer database from the American College of Surgeons website. Due to the retrospective and de-identified nature of the study, the study’s protocol was approved by Cleveland Clinic’s Institutional Review Board (IRB) as Exempt Human Subject Research (IRB #22–153). The waiver of informed consent to participate and publish the data in an online open-access publication was granted in the application due to minimal risk research involving human subjects. This study was conducted in accordance with the regulations of the ethical review committee and the guidelines of the journal.

### Patient selection

Patients included were diagnosed with non-metastatic resectable gastric and GEJ cancer between 2010 and 2020 with a cT2 stage. Given the importance of the quality of surgery on outcomes, only patients who underwent gastrectomy/esophagectomy with lymph node dissection (LND) were included. Patients who received immunotherapy/hormonal therapy were excluded due to the underlying effects of these therapies on outcomes, and those with metastatic (cM1), unknown staging, and non-adenocarcinoma histology were excluded. Since the NCDB does not provide a single variable to describe the extent of LND, the variable “regional lymph nodes examined” done by a pathologist after surgery was used to approximate the extent of dissection. As stated by the National Comprehensive Cancer Network (NCCN), an adequate D2 lymphadenectomy includes the removal of more than 15 lymph nodes during surgery. NCCN also recommends performing D2 LND at high-volume gastric surgery centers. For this reason, only patients who had > 15 LND and were treated at academic, comprehensive community, or integrated cancer centers were included. Moreover, since most chemotherapy regimens approved for treating GC include multiple agents, only patients who received multi-agent chemotherapy were included.

### Demographic and clinical variables

Socio-demographic (age, race, ethnicity, year of diagnosis, facility type, and insurance type), clinic-pathological characteristics (Charlson-Deyo comorbidity score, histology, grade, primary site of tumor, cT and cN stage, positive regional lymph nodes), and treatment combination were evaluated in the analysis. Patients were classified into different groups based on the sequence of administration of multiagent chemotherapy and radiotherapy. The treatment modalities of interest were NCR, NC, PC, AC, and ACR. A subgroup of patients with GEJ tumors was separately identified using the International Classification of Disease primary site classification “C16.0” and NCDB’s “site-specific factor 25”, which discriminates the tumor’s location with respect to GEJ.

### Statistical analysis

Data analysis was conducted using the Statistical Package for the Social Sciences (SPSS), version 28.0. The chi-square test evaluated significant socio-demographic and clinical characteristics differences between treatment cohorts among the total population. In addition, a multivariable Cox regression analysis with backward elimination was utilized to explore the association of independent survival factors for the total population and patients with GEJ separately. Kaplan–Meier (KM) curve compared OS between the different treatment modalities. A Cochran-Armitage test was also utilized to evaluate the treatment trends over our study period. The median follow-up period for the survival analysis is 31.51 months (IQR 18.30–54.60). Two-sided p-values < 0.05 were considered statistically significant.

## Results

### Socio-demographic and clinical characteristics

We identified 7,665 patients with non-metastatic ^2^cT2 gastric and GEJ tumors. The majority of patients fell in the NCR group (*n* = 2724, 36.1%), followed by the NC group (*n* = 2157, 28.5%) and the PC group (*n* = 1532, 20.3%). The cohort was comprised primarily of males (70.3%) with a median age of 63 (IQR 56–75). Moreover, there was a predominance of Caucasian/White racial group (75.5%) and non-Hispanic ethnicity (88.1%). A significant proportion of tumors were located in the gastric cardia/GEJ (48.3%), had a T3 clinical staging (67.9%) and poorly differentiated grade (61.8%), and were mainly treated at an academic/research cancer program (CP) (57.7%). Interestingly, most patients who received NCR had tumors located in the GEJ (94.7%), while the majority of patients who received PC had tumors located in the stomach (78.5%). The results of the demographic and clinical characteristics of each treatment arm are summarized in Tables 1 and 2.

**Table 1:** Chi-square analysis of the demographic and clinical characteristics of patients in each treatment cohort.

Variable	Total *N* = 7,665	NCR^a^ *n* = 2,724	NC^b^ *n* = 2,157	PC^c^ *n* = 1,532	ACR^d^ *n* = 525	AC^e^ *n* = 727	*p*-value
Age, n (%)							< 0.0001
18–56	1840 (24.0)	577 (21.2)	477 (22.1)	418 (27.3)	173 (33.0)	195 (26.8)	
56–63	1698 (22.2)	683 (25.1)	414 (19.2)	366 (23.9)	91 (17.3)	144 (19.8)	
63–70	1984 (25.9)	782 (28.7)	543 (25.2)	374 (24.4)	122 (23.2)	163 (22.4)	
≥70	2143 (27.9)	682 (25.0)	723 (33.5)	374 (24.4)	139 (26.5)	225 (31.0)	
Sex, n (%)							< 0.0001
Male	5391 (70.3)	2290 (84.1)	1360 (63.1)	966 (63.1)	338 (64.4)	437 (60.1)	
Female	2274 (29.7)	434 (15.9)	797 (36.9)	566 (36.9)	187 (35.6)	290 (39.9)	
Race, n (%)							< 0.0001
White	5747 (75.5)	2545 (93.7)	1447 (67.6)	981 (64.8)	317 (60.7)	457 (63.4)	
Black	905 (11.9)	90 (3.3)	369 (17.3)	237 (15.6)	102 (19.6)	107 (14.9)	
Other	959 (12.6)	81 (3.0)	322 (15.1)	297 (19.6)	103 (19.7)	156 (21.7)	
Hispanic, n (%)							< 0.0001
Yes	909 (11.9)	108 (4.0)	328 (15.2)	262 (17.1)	80 (15.2)	131 (18.0)	
No	6756 (88.1)	2616 (96.0)	1829 (84.8)	1270 (82.9)	445 (84.8)	596 (82.0)	
Year of diagnosis, n (%)							< 0.0001
2010–2011	836 (10.9)	267 (9.8)	198 (9.2)	137 (8.9)	130 (24.8)	104 (14.3)	
2012–2013	1037 (13.5)	381 (14.0)	276 (12.8)	130 (8.5)	136 (25.9)	114 (15.7)	
2014–2015	1258 (16.4)	540 (19.8)	323 (15.0)	183 (12.0)	93 (17.7)	119 (16.3)	
2016–2017	1656 (21.6)	653 (24.0)	437 (20.2)	331 (21.6)	84 (16.0)	151 (20.8)	
2018–2020	2878 (37.6)	883 (32.4)	923 (42.8)	751(49.0)	82 (15.6)	239 (32.9)	
Insurance, n (%)							< 0.0001
Uninsured	240 (3.1)	46 (1.7)	73 (3.4)	58 (3.8)	34 (6.5)	29 (4.0)	
Private	3501 (45.7)	1334 (49.0)	868 (40.2)	764 (49.9)	232 (44.2)	303 (41.7)	
Medicaid/other government	783 (10.2)	195 (7.1)	239 (11.1)	177 (11.5)	64 (12.2)	108 (14.9)	
Medicare	3141 (41.0)	1149 (42.2)	977 (45.3)	533 (34.8)	195(37.1)	287(39.4)	
Facility type, n (%)							< 0.0001
Comprehensive Community CP^f^	1980 (25.8)	674 (24.8)	492 (22.8)	391 (25.5)	196 (37.3)	227 (31.2)	
Academic Cancer Program	4419 (57.7)	1540 (56.5)	1316 (61.0)	934 (61.0)	235 (44.8)	394 (54.2)	
Integrated Network CP^f^	1266 (16.5)	510 (18.7)	349 (16.2)	207 (13.5)	94 (17.9)	106 (14.6)	
CCI, n (%)							0.4060
0	5471 (71.4)	1954 (71.7)	1497 (69.4)	1109 (72.3)	383 (73.0)	528 (72.6)	
1	1544 (20.1)	539 (19.8)	470 (21.8)	301 (19.7)	102 (19.4)	132 (18.2)	
2/3	650 (8.5)	231 (8.5)	190 (8.8)	122 (8.0)	40 (7.6)	67 (9.2)	

^a^NCR, Neoadjuvant Chemoradiotherapy; ^b^NC, Neoadjuvant Chemotherapy; ^c^PC, Perioperative Chemotherapy; ^d^ACR, Adjuvant Chemoradiotherapy; ^e^AC, Adjuvant Chemotherapy; ^f^CP, Cancer Program; CCI, Charlson Comorbidity Index.

**Table 2:** Chi-square analysis of the clinical and pathological tumor characteristics of patients in each treatment cohort.

Variable	Total *N* = 7665	NCR^a^ *n* = 2724	NC^b^ *n* = 2157	PC^c^ *n* = 1532	ACR^d^ *n* = 525	AC^e^ *n* = 727	*p*-value
Clinical T-stage, n (%)							< 0.0001
2	1647 (22.8)	472 (17.6)	484 (23.4)	348 (23.5)	138 (32.4)	205 (36.1)	
3	4903 (67.9)	2116 (79.0)	1338 (64.7)	968 (65.2)	197 (46.4)	284 (50.0)	
4	674 (9.3)	91 (3.4)	246 (11.9)	168 (11.3)	90 (21.2)	79 (13.9)	
Clinical N-stage, n (%)							< 0.0001
0	3156 (43.2)	1002 (37.2)	946 (45.4)	651 (43.5)	218 (49.8)	339 (57.1)	
1	2764 (37.8)	1183 (43.9)	793 (38.0)	549 (36.8)	104 (23.7)	135 (22.8)	
2	1105 (15.1)	448 (16.6)	284 (13.6)	246 (16.5)	54 (12.3)	73 (12.3)	
3	281 (3.9)	62 (2.3)	63 (3.0)	48 (3.2)	62 (14.2)	46 (7.8)	
Grade, n (%)							< 0.001
Well-Differentiated	236 (3.1)	115 (4.2)	66 (3.0)	26 (1.7)	16 (3.0)	13 (1.8)	
Moderately Differentiated	2118 (27.6)	1025 (37.6)	509 (23.6)	336 (21.9)	107 (20.4)	141 (19.4)	
Poorly Differentiated	4733 (61.8)	1422 (52.2)	1397 (64.8)	1036 (67.6)	377 (71.8)	501 (68.9)	
Unknown	578 (7.5)	162 (6.0)	185 (8.6)	134 (8.8)	25 (4.8)	72 (9.9)	
Tumor size, n (%)							< 0.0001
< 27 mm	1437 (23.4)	575 (27.4)	406 (24.2)	275 (23.0)	73 (14.7)	108 (16.2)	
27–40 mm	1201 (19.6)	435 (20.7)	292 (17.4)	243 (20.3)	90 (18.1)	141 (21.3)	
40–60 mm	1708 (27.8)	606 (28.8)	470 (28.0)	323 (27.0)	144 (29.0)	165 (24.9)	
> 60 mm	1789 (29.2)	485 (23.1)	509 (30.4)	356 (29.7)	190 (38.2)	249 (37.6)	
Nodes positive, n (%)							< 0.0001
0	3460 (45.2)	1567 (57.6)	1019 (47.3)	656 (42.8)	78 (14.8)	140 (19.3)	
1–2	1508 (19.7)	592 (21.8)	399 (18.5)	287 (18.7)	89 (17.0)	141 (19.5)	
3–10	1752 (22.9)	454 (16.7)	474 (22.0)	368 (24.0)	218 (41.5)	238 (32.8)	
≥ 11	934 (12.2)	105 (3.9)	262 (12.2)	221 (14.5)	140 (26.7)	206 (28.4)	
Histology, n (%)							< 0.0001
Adenocarcinoma, not specified	5032 (65.7)	2358 (86.6)	1217 (56.4)	821 (53.6)	255 (48.6)	381 (52.4)	
Diffuse type	383 (5.0)	11 (0.4)	139 (6.4)	106 (6.9)	60 (11.4)	67 (9.2)	
Intestinal type adenocarcinoma	698 (9.1)	62 (2.3)	288 (13.4)	193 (12.6)	59 (11.2)	96 (13.2)	
Mucinous adenocarcinoma	136 (1.8)	51 (1.9)	34 (1.6)	20 (1.3)	20 (3.8)	11 (1.5)	
Signet ring cell carcinoma	1365 (17.8)	224 (8.2)	461 (21.4)	387 (25.3)	127 (24.2)	166 (22.8)	
Carcinoma/undifferentiated/mixed	51 (0.6)	18 (0.6)	18(0.8)	5 (0.3)	4 (0.8)	6 (0.9)	
Primary site, n (%)							< 0.0001
Gastroesophageal junction	3703 (48.3)	2579 (94.7)	547 (25.3)	330 (21.5)	122 (23.2)	125 (17.2)	
Fundus of stomach	227 (3.0)	16 (0.6)	99 (4.6)	68 (4.4)	17 (3.2)	27 (3.7)	
Body of stomach	629 (8.2)	22 (0.8)	275 (12.8)	201 (13.1)	48 (9.1)	83 (11.4)	
Gastric antrum	1094 (14.3)	24 (0.9)	425 (19.7)	329 (21.5)	138 (26.3)	178 (24.5)	
Pylorus	169 (2.2)	6 (0.2)	59 (2.7)	56 (3.7)	24 (4.6)	24 (3.3)	
Lesser curvature of stomach	611 (7.9)	17 (0.6)	250 (11.6)	208 (13.6)	63 (12.0)	73 (10.0)	
Greater curvature of stomach	207 (2.7)	8 (0.3)	81 (3.8)	63 (4.1)	15 (2.9)	40 (5.5)	
Overlapping lesion of stomach	572 (7.5)	27 (1.0)	227 (10.5)	182 (11.9)	47 (9.0)	89(12.2)	
Stomach, not specified	453 (5.9)	25 (0.9)	194 (9.0)	95 (6.2)	51 (9.7)	88(12.1)	
Margin status, n (%)							< 0.0001
No residual tumor	6870 (89.6)	2531 (92.9)	1926 (89.2)	1391 (90.8)	426 (81.1)	596 (82.0)	
Residual tumor, not specified	296 (3.9)	73 (2.7)	91 (4.2)	42 (2.7)	35 (6.7)	55 (7.6)	
Microscopic residual tumor	399 (5.2)	93 (3.4)	109 (5.1)	82 (5.3)	55 (10.5)	60 (8.2)	
Macroscopic residual tumor	26 (0.3)	5 (0.2)	8 (0.4)	4 (0.3)	1 (0.2)	8 (1.1)	
Unknown applicable/indeterminate	74 (1.0)	22 (0.8)	23 (1.1)	13 (0.9)	8 (1.5)	8 (1.1)	
Lymph-vascular inv, n (%)							< 0.0001
Not present	3429 (44.7)	1455 (53.4)	963 (44.6)	628 (41.0)	160 (30.5)	223 (30.7)	
Present	2613 (34.1)	567 (20.8)	765 (35.5)	554 (36.2)	295 (56.2)	432 (59.4)	
Unknown	1623 (21.2)	702 (25.8)	429 (19.9)	350 (22.8)	70 (13.3)	72(9.9)	

^a^NCR, Neoadjuvant chemoradiotherapy; ^b^NC, Neoadjuvant chemotherapy; ^c^PC, Perioperative chemotherapy; ^d^ACR, Adjuvant chemoradiotherapy; ^e^AC, Adjuvant chemotherapy.

### Survival among the different treatment modalities

**Fig. 1:**
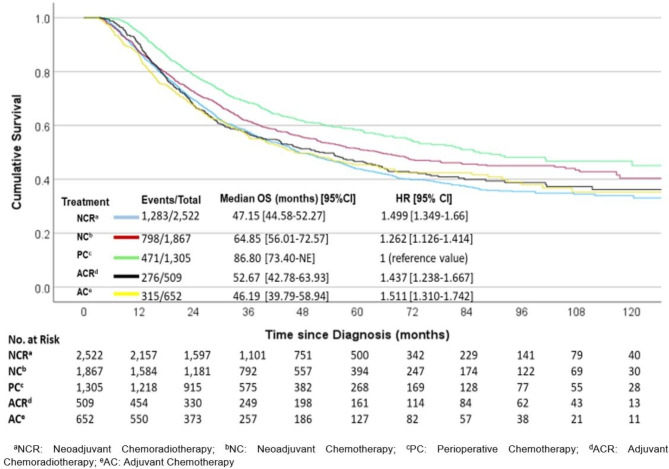
Kaplan–Meier curve of OSl for the different treatment modalities among the total population.

Patients undergoing PC had the most prolonged OS (median 86.80 months, 95% CI 73.40-NE), followed by NC (median 64.85 months, 95% CI 56.02–72.57). In contrast, those undergoing AC had the worst OS (median 46.19 months, 95% CI 39.79–58.94, log-rank p-value < 0.001), as reported in Fig. 1. Patients who received chemoradiotherapy in the neoadjuvant and adjuvant settings had worse median OS than PC and NC (NCR 47.15 months, 95% CI 44.58–52.27 and ACR 52.67 months, 95% CI 42.78–63.93). Moreover, Table 3 shows the multivariable Cox regression analysis, where NCR and NC had worse survival compared to the PC (HR 1.74, 95% CI 1.50–2.02, *p* < 0.001 and HR 1.26, 95%C I 1.10–1.44, *p* = 0.0008, respectively). OS follow-up was conducted at 1-year, 2-year, and 5-years intervals (Supplementary Table 1).

**Table 3:** Multivariable Cox regression analysis for OS of the total population.

Variable	HR (95% CI)	*p*-value
Treatment sequence		
Perioperative chemotherapy	1	-
Neoadjuvant chemoradiotherapy	1.74 (1.50–2.02)	< 0.0001
Adjuvant chemoradiotherapy	0.91 (0.77–1.09)	0.3238
Adjuvant chemotherapy	1.01 (0.85–1.21)	0.8831
Neoadjuvant chemotherapy	1.26 (1.10–1.44)	0.0008
Age		
<56	1	-
56–63	1.13 (1.00-1.29)	0.0449
63–70	1.19 (1.05–1.34)	0.0049
>70	1.46 (1.30–1.64)	< 0.0001
Race		
White	1	-
Black	1.01 (0.87–1.16)	0.9396
Other	0.60 (0.51–0.71)	< 0.0001
Hispanic vs. non-Hispanic (ref.)	0.80 (0.69–0.92)	0.0026
Year of diagnosis		
2018–2020	1	-
2010–2011	1.41 (1.21–1.63)	< 0.0001
2012–2013	1.20 (1.04–1.39)	0.0127
2014–2015	1.20 (1.04–1.38)	0.0128
2016–2017	1.07 (0.93–1.23)	0.3399
CCI		
0	1	-
1	1.12 (1.01–1.23)	0.0325
2/3	1.25 (1.08–1.45)	0.0032
Facility type		
Academic/research program	1	-
Comprehensive community CP	1.18 (1.07–1.30)	0.0010
Integrated network cancer program	1.21 (1.09–1.36)	0.0007
Grade		
Well differentiated	1	-
Moderately differentiated	0.89 (0.70–1.14)	0.3558
Poorly differentiated/undifferentiated	1.11 (0.88–1.41)	0.3856
Clinical T stage		
2	1	-
3	1.26 (1.13–1.41)	< 0.0001
4	1.28 (1.08–1.51)	0.0037
Histology		
Adenocarcinoma, not specified	1	-
Diffuse type	1.22 (1.00-1.49)	0.0502
Intestinal type adenocarcinoma	0.83 (0.70-1.00)	0.0492
Mucinous adenocarcinoma	0.75 (0.57–0.98)	0.0350
Signet ring cell carcinoma	1.22 (1.08–1.36)	0.0008
Carcinoma-undifferentiated/mixed	2.34 (1.59–3.44)	< 0.0001
Margin status		
No residual tumor	1	-
Residual tumor, not specified	1.54 (1.29–1.84)	< 0.0001
Microscopic residual tumor	1.71 (1.46–1.99)	< 0.0001
Macroscopic residual tumor	1.79 (0.92–3.46)	0.0862
Unknown/not applicable	1.35 (0.94–1.93)	0.1037
Lymph-vascular invasion		
Not present	1	-
Present	1.29 (1.17–1.43)	< 0.0001
Unknown	0.99 (0.87–1.11)	0.8212
Primary site		
Gastroesophageal junction	1	-
Fundus of stomach	1.41 (1.12–1.79)	0.0039
Body of stomach	0.84 (0.70–1.02)	0.0759
Gastric antrum	0.72 (0.61–0.85)	0.0001
Pylorus	0.68 (0.48–0.97)	0.0345
Lesser curvature of stomach	0.70 (0.57–0.85)	0.0004
Greater curvature of stomach	0.67 (0.50–0.89)	0.0061
Overlapping lesion of stomach	0.99 (0.82–1.18)	0.8674
Stomach, not otherwise specified	1.13 (0.94–1.37)	0.2006
Positive regional nodes		
0	1	-
1–2	1.60 (1.42–1.81)	< 0.0001
3–10	2.41 (2.14–2.71)	< 0.0001
≥11	4.06 (3.51–4.68)	< 0.0001
Tumor size		
< 27 mm	1	-
27–40 mm	1.02 (0.89–1.16)	0.7963
40–60 mm	1.10 (0.97–1.24)	0.1470
> 60 mm	1.21 (1.07–1.36)	0.0028

All variables in Tables 1 and 2 were included in the Cox model as explanatory variables, and finally, clinical N-stage, insurance status, and sex were eliminated by the backward elimination method (*p* > 0.1).

Furthermore, tumors located in the fundus of the stomach had lower survival compared to proximal tumors (HR 1.41, 95% CI 1.12–1.79, *p* = 0.0039). Patients diagnosed during 2010–2011 had a worse prognosis than patients diagnosed in 2018–2020 (HR 1.41, 95% CI 1.21–1.63, *p* < 0.0001).

In addition, integrated network and comprehensive community CP performed worse than academic CP (HR 1.21, 95% CI 1.09–1.36, *p* = 0,0007 and HR 1.18, 95% CI 1.07–1.30, *p* = 0.0010).

### Survival outcomes in patients with GEJ tumors

In patients with GEJ tumors, the PC group performed better than the NCR (HR 1.69, 95% CI 1.35–2.11, *p* < 0.001). Hispanic ethnicity had better OS than non-Hispanic ethnicity (HR 0.68, 95% CI 0.49–0.93, p 0.0161). Table 4 describes significant factors associated with OS for patients with GEJ tumors in a multivariable Cox regression model.

**Table 4:** Multivariable Cox regression analysis for OS for patients with GEJ tumors only.

Variable	HR (95% CI)	*p*-value
Treatment sequence		
Perioperative chemotherapy	1	–
Neoadjuvant chemoradiotherapy	1.69 (1.35–2.11)	< 0.0001
Adjuvant chemoradiotherapy	1.06 (0.76–1.49)	0.7171
Adjuvant chemotherapy	1.06 (0.74–1.51)	0.7672
Neoadjuvant chemotherapy	1.16 (0.90–1.50)	0.2582
Hispanic vs. non-hispanic	0.68 (0.49–0.93)	0.0161
Insurance status	0.84 (0.54–1.33)	0.4671
Private		
Uninsured	1	–
Medicaid/other government	1.14 (0.90–1.44)	< 0.0001
Medicare	1.32 (1.18–1.48)	0.2770
CCI^a^		
0	1	–
1	1.15 (1.01–1.32)	0.0378
2/3	1.26 (1.04–1.53)	0.0212
Grade		
Well differentiated	1	–
Moderately differentiated	0.90 (0.68–1.19)	0.4602
Poor/Undifferentiated	1.22 (0.93–1.61)	0.1551
Clinical T-stage		
2	1	–
3	1.38 (1.18–1.60)	< 0.0001
4	1.26 (0.92–1.73)	0.1487
Nodes positive		
0	1	–
1–2	1.59 (1.38–1.84)	< 0.0001
3–10	2.30 (1.98–2.67)	< 0.0001
≥11	3.18 (2.55–3.97)	< 0.0001
Histology		
Adenocarcinoma	1	–
Diffuse type	0.85 (0.42–1.74)	0.6607
Intestinal type adenocarcinoma	0.97 (0.69–1.36)	0.8606
Mucinous adenocarcinoma	0.80 (0.55–1.15)	0.2253
Signet ring cell carcinoma	1.06 (0.89–1.27)	0.4974
Carcinoma-undifferentiated/mixed	2.65 (1.73–4.08)	< 0.0001
Margin status		
No residual tumor	1	–
Residual tumor, not specified	1.55 (1.17–2.05)	0.0022
Microscopic residual tumor	1.63 (1.30–2.04)	< 0.0001
Macroscopic residual tumor	1.26 (0.52–3.05)	0.6086
Unknown/not applicable/indeterminate	1.32 (0.74–2.34)	0.3491
Lymph-vascular invasion		
Not present	1	–
Present	1.32 (1.16–1.51)	< 0.0001
Unknown	0.97 (0.84–1.13)	0.7125

^a^CCI, Charlson Comorbidity Index. All variables in Tables 1 and 2 were included in the Cox model as explanatory variables, and finally, race, tumor size, sex, year of diagnosis, facility type, age, and clinical N-stage were eliminated by the backward elimination method (*p* > 0.1).

### Trends in treatment modalities

**Fig. 2:**
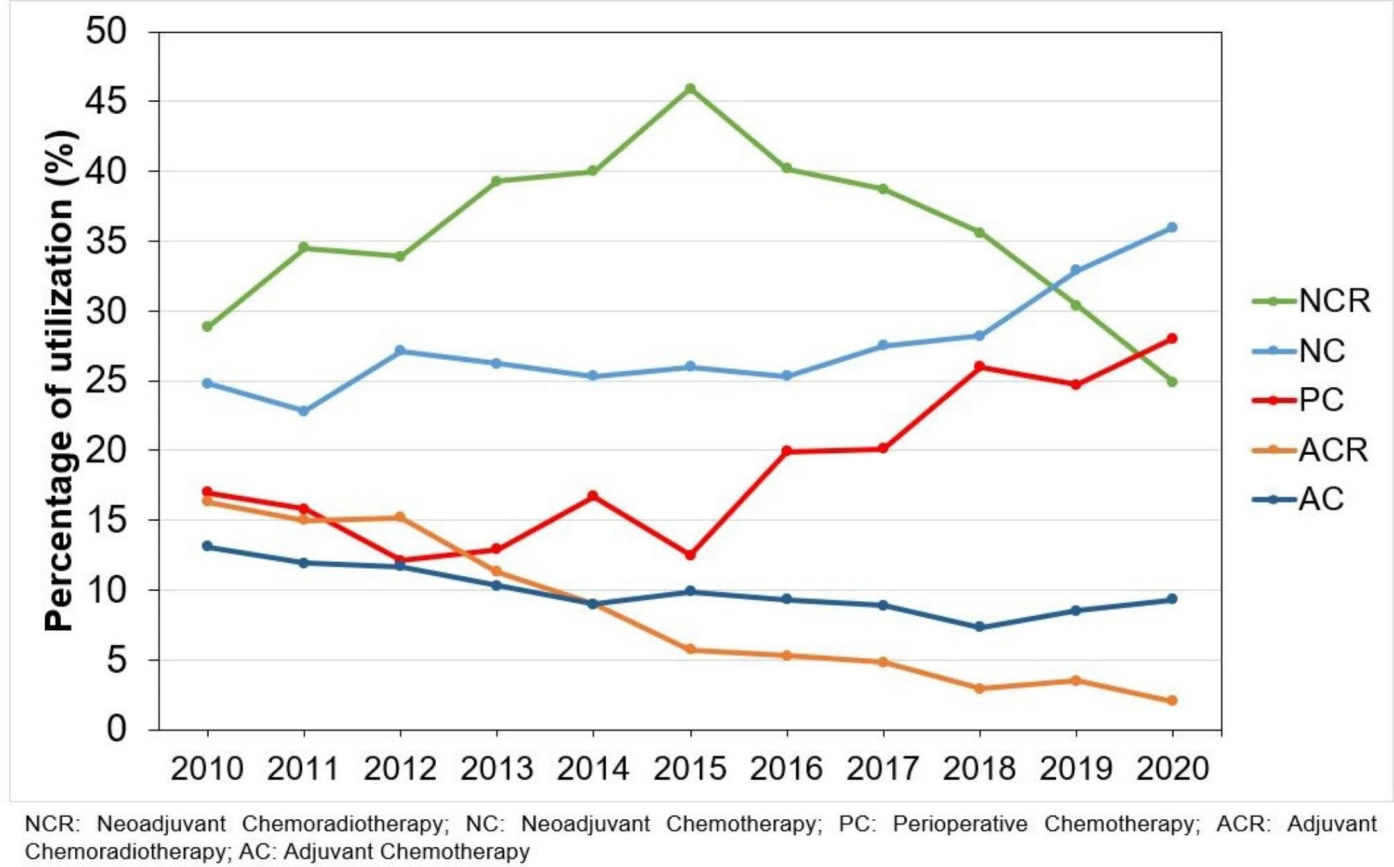
Linear graph depicting the utilization trend of the different treatment modalities per year of diagnosis.

As depicted in Fig. 2, both NC and the PC approach recorded the highest increase in utilization between 2010 and 2020 (11.0%). In 2010, NCR (28.8%) was the most utilized modality, followed by NC (24.8%), while PC utilization was 17.0%. Our results demonstrate a peak increase in utilization of NCR between 2010 and 2015, reaching 45.9%. In 2020, NCR utilization decreased to 24.9% (3rd most utilized), while the most used modality was NC (35.8%), followed by PC (28.0%). Meanwhile, ACR and AC fell out of favor, gradually declining to reach 2.0% and 9.3%, respectively.

## Discussion

To our knowledge, this is the most extensive and most recent national retrospective analysis exploring treatment patterns and survival outcomes for patients with resectable non-metastatic gastric and GEJ cancer. Data reported in the NCDB reflect real-world trends and outcomes at various treatment facilities across the US and are essential to highlight practice quality in research and non-research settings.

Despite several trials in the recent past, there is still a need to establish a global standard of care through multicenter head-to-head trials. Additionally, the role of radiation therapy has been questioned with the integration of extended D2 lymphadenectomy and the introduction of multi-agent chemotherapy agents in the preoperative and postoperative settings. In our analysis, we report the association of the PC with improved OS in patients with gastric and GEJ cancer. We also reveal a correlation with suboptimal outcomes in comparison to PC for patients who received chemoradiotherapy in the neoadjuvant and adjuvant settings. Even with a significant positive trend in the utilization of PC and NC and evidence of benefit in the literature, the percentage of utilization for PC remains low. Here, we discuss the advantages and limitations of the evaluated treatment options and explore reasons for shifting trends in utilization.

The evidence supporting PC efficacy for gastric and GEJ cancers is rooted in well-designed clinical trials. The MAGIC trial demonstrated a better OS and an increase in 5-year survival (36% vs. 23%) in patients receiving perioperative epirubicin, cisplatin, and fluorouracil (ECF) over surgery alone (HR 0.75, *p* = 0.009)^8^. The German FLOT-4 trial, published in 2017, subsequently compared docetaxel, oxaliplatin, leucovorin, and fluorouracil (FLOT regimen) to the control group ECF, with improved R0 resection margins and survival for the FLOT regimen^9^. Additional studies reinforced the efficacy of PC across different geographic and patient cohorts, such as the French FNCLCC/FFCD trial^10^ and another trial conducted in Korea^11^. These trials have been instrumental in creating a paradigm shift towards PC for patients with GC, with US and European guidelines adopting both regimens as recommended options with the highest level of evidence^5,6^. Additionally, our results report the association of PC with improved outcomes in patients with GC and GEJ tumors.

Nonetheless, it is essential to note that out of all patients randomized in the German trial, only 46% in the perioperative FLOT group and 37% of patients in the perioperative ECF/ECX control group completed the treatment, primarily because of toxicity, patient requests, and disease progression^9^. Meanwhile, 104 of the 250 patients randomly assigned to the PC ECF group in the MAGIC completed all six cycles of chemotherapy preoperatively and postoperatively (41.6%)^8^. Interestingly, almost 50% of patients who completed preoperative and surgery protocols could not complete the postoperative regimen for the previously mentioned reasons. This poses the issue of the adequacy of completion and tolerating a high-intensity chemotherapy approach. The low postoperative completion rate emphasizes the need to compare neoadjuvant with perioperative approaches in prospective studies and optimize patient selection for either treatment modality, especially in patients expected not capable of completing their postoperative regimen due to surgical complications or toxicity events. Our analysis shows that NC had the highest utilization rate during 2020 (35.8%), followed by PC (28.0%). While it is plausible that the slow acceptance of PC as the standard of care is due to the relative novelty of the published trials, it is also highly likely that patients were not capable of completing postoperative chemotherapy course, leading to a higher percentage of patients receiving NC than PC.

Alternatively, guidelines recommending NCR for patients with GC, with a lower category of evidence, are mainly derived from trials on patients with esophageal and GEJ tumors, such as the notable Dutch CROSS trial^12^. The trial, published in 2012, demonstrated superior survival outcomes with NCR over surgery alone, establishing NCR as the preferred treatment practice for patients with esophageal cancer and GEJ tumors (Siewert type I and II). Nevertheless, no prospective data compared PC with NCR for patients with GC alone. Additionally, the efficacy of NCR - in both gastric and GEJ cancer - is debatable in comparison to NC treatment modalities due to its lack of inclusion in prospective studies. The POET trial was one of the few trials directly comparing NCR and NC only in patients with GEJ tumors^13^. While OS was skewed toward NCR intervention (HR 0.65, p 0.055), it failed to reach statistical significance. Two meta-analyses that included randomized controlled studies showed better pathological and survival outcomes with NCR over NC in patients with GC^14,15^. However, the type of surgery and the extent of LND were not part of the inclusion criteria, significantly downplaying radiation’s therapeutic role. In our adjusted survival analysis, NCR was still associated with worse survival than PC and NC, suggesting that the therapeutic role of radiation with extended LND may be disadvantageous. Our results of treatment patterns show a peak increase in utilization for NCR between 2010 and 2015, as evident in Fig. 2 (17.1%), in accordance with the period during which the CROSS trial was published.

Although GEJ tumors are recording an increase in incidence^16^ and most of our population presented with proximal tumors, the number of trials emphasizing GEJ tumor treatment options is still insufficient, as these patients were inconsistently included in either gastric or esophageal cancer trials. Evidence used to guide multimodality treatment of GEJ is extrapolated from trials on gastric or esophageal malignancies, and there was a lack of standardized inclusion criteria. NCR is considered the standard approach for GEJ tumors, as it demonstrated a survival advantage over surgery alone, and 24% of randomized patients in the CROSS trial had a GEJ tumor^8^. Interestingly, our results show that around 95% of patients who received NCR were diagnosed with GEJ tumors, indicating a strong adherence to the standard of care treatment for GEJ tumors. Nevertheless, recent results of the long-awaited ESOPEC trial demonstrated superior survival outcomes for the perioperative FLOT regimen in comparison to the NCR CROSS regimen in patients with GEJ cancer^17^. This evidence is supported by the FNCLCC/FFCD trial results that showed significant PC effects were more evident for GEJ cancer (as a subgroup analysis) and less for stomach cancer in comparison to surgery alone^10^. Furthermore, 55.6% of patients included in the FLOT-4 trial had tumors in the GEJ (Siewert types I, II, and III), and the inclusion of these patients helped the authors conclude that perioperative FLOT may be effective for Siewert type I and II, classically treated as esophageal tumors^9^. Given the potential “practice-changing” results of the ESOPEC trial and earlier evidence of PC efficacy in GEJ tumors, a major shift in treatment paradigms for patients with GEJ may be necessary. On the other hand, other studies, including the MAGIC trial, failed to show a favorable response in GEJ patients with PC^8^. Our subgroup analysis of GEJ tumors revealed an association between PC and improved OS in comparison to NCR/NC.

The role of the AC approach is primarily accepted in Eastern countries and is often used for patients who did not receive preoperative chemotherapy and radiation. The ARTIST and CLASSIC trials carried out in Asia on patients who had a gastrectomy and a D2 lymphadenectomy showed the benefit of adjuvant capecitabine and cisplatin/oxaliplatin combination over surgery alone^18,19^. The addition of radiotherapy in the adjuvant setting in the ARTIST and ARTIST-II trials failed to show a significant difference in outcomes, even in subgroups of patients with pathologically staged lymph node-positive cancer^18,20^. Head-to-head comparisons of AC and PC were performed in both the RESOLVE and PRODIGY trials^11,21^. While different chemotherapy combinations were utilized in the preoperative and postoperative settings, both demonstrated the PC approach’s superiority, similar to our analysis results. Despite the multivariable analysis not showing a significant difference in OS between PC and adjuvant therapies, our unadjusted survival analysis showed that PC was associated with superior OS to ACR and AC. In terms of utilization, AC and ACR recorded a decreasing trend during our study (13.1–9.3% for AC and 16.3–2.0% for ACR) due to their lack of popularity in the West. Apart from the “Macdonald” trial (published in 2001), most of the trials for adjuvant treatments were conducted in the East, while neoadjuvant therapies have been gaining traction during the last decade, given the established benefits of neoadjuvant therapies^22^.

Figure 2 portrays the percentage of utilization rate for each treatment regimen per year of diagnosis. The most utilized treatment modality during 2010 for all patients, including patients with GEJ tumors, was NCR (28.8%), followed by NC (24.8%). In the most recent year of our study (2020), NC (35.8%) became the most utilized, followed by PC (28.0%) and NCR (24.9%). We demonstrated the highest increase in utilization for PC and NC (11.0% for both modalities) from 2010 to 2020. While the percentage of patients undergoing PC is increasing and national guidelines recommend PC with the highest level of evidence, real-world data in the NCDB from high-volume centers still reveals a low utilization rate. After the release of the FLOT-4 trial results in 2017, there was a 6% increase in PC utilization between 2016 and 2017, the highest rate per year among all treatment modalities. Simultaneously, NC recorded a 10% increase from 2016 to 2020. The increase in utilization of NC can be attributed to the increased acceptance of PC, which includes preoperative systemic treatment, but patients could not tolerate postoperative treatment. Similar to the landmark FLOT-4 and MAGIC trials^8,9^, our results show that while PC is promising, there is a high discontinuation rate, emphasizing the need to evaluate alternative preoperative chemotherapy regimens complemented by immunotherapy, targeted, and hormonal therapy.

The study’s retrospective nature limits the ability to control for unobservable confounding factors. Plus, data is extrapolated from the NCDB, which collects pre-designated variables from CoC- (Commission of Cancer) accredited institutions, restricting the ability to report reasons for not receiving adjuvant treatment. Many patients may have opted out due to intolerable adverse events, postoperative complications, or patient/clinician decisions. Another limitation of the NCDB is that the regimen or dosing of chemotherapy agents administered is not stated. Thus, optimal chemotherapeutic agents could not be concluded. Additionally, survival metrics other than overall survival, such as disease-free survival and progression-free survival, are not reported.

## Conclusion

Real-world survival outcomes from the NCDB reveal the correlation of PC treatment with improved outcomes over other acceptable modalities for patients with nonmetastatic gastric and GEJ cancer. Despite its positive trend over the years, the results of this nationwide study reveal significant underutilization of PC, which condones the need to improve efforts to enhance adherence to national recommendations or provide alternative tolerable regimens. Patients who received combination therapy that included radiation with extended LND had a worse prognosis compared to patients who received chemotherapy. Clinical trials and meta-analyses would aid in confirming the optimal combination regimen with careful consideration of the toxicity profile to ascertain its tolerability and decrease disease burden. Finally, promising immunotherapies and targeted therapies currently under investigation may pave the way for unprecedented success in outcomes.

## Electronic supplementary material

Below is the link to the electronic supplementary material.

Supplementary Material 1

## Data Availability

The data supporting this study’s findings are available for investigators who are granted access to the National Cancer Database through an online application from the ACS website [https://www.facs.org/quality-programs/cancer-programs/national-cancer-database/puf/]. The participant user files (PUF) for the pancreatic cancer database were downloaded by the principal investigator associated with a Commission on Cancer (CoC) accredited institution.
