# Supplementary material for: Evaluating survival outcomes and treatment recommendations in resectable gastric cancer
Source: Sci Rep. 2025 Jan 22;15:2816. doi: 10.1038/s41598-024-82807-8 (PMC11754744; doi:10.1038/s41598-024-82807-8)
Supplement: Supplementary file 1 — Supplementary Material 1 [file 41598_2024_82807_MOESM1_ESM.docx]

**Survival Outcomes in Resectable Gastric Cancer: A Comprehensive Evaluation of Recommended Treatment Options**

Original Article

Saad Sabbagh, M.D.^1*^; Iktej Singh Jabbal, M.D.^2^; María Herrán, M.D.^1^; Mohamed Mohanna, M.D.^1^; Sindu Iska, M.D.^1^; Mira Itani, M.D.^1^; Barbara Dominguez, M.B.A.^1^; Kaylee Sarna, M.S.^3^; Zeina Nahleh, M.D., F.A.C.P.^1^; Arun Nagarajan, M.D.^1^

^1^Department of Hematology-Oncology, Maroone Cancer Center, Cleveland Clinic Florida, Weston, FL, U.S.A

^2^Department of Internal Medicine, Advent Health Sebring, Sebring, FL, U.S.A

^3^Department of Clinical Research, Cleveland Clinic Florida, Weston, FL, U.S.A

**Supplementary Table 1.** 1-year, 2-year, and 5-year Follow-up for Overall Survival Outcomes Stratified by Treatment.

| **Treatment** | **1-year OS (%)** | **2-year OS (%)** | **5-year OS (%)** |
| --- | --- | --- | --- |
| **Neoadjuvant Chemoradiotherapy** | 87.2 | 69.7 | 43.9 |
| **Neoadjuvant Chemotherapy** | 87.7 | 72.7 | 51.5 |
| **Perioperative Chemotherapy** | 94.8 | 78.8 | 58.4 |
| **Adjuvant Chemoradiotherapy** | 90.3 | 67.7 | 46.6 |
| **Adjuvant Chemotherapy** | 86.4 | 67.8 | 45.4 |
